# Comparative and functional triatomine genomics reveals reductions and expansions in insecticide resistance-related gene families

**DOI:** 10.1371/journal.pntd.0005313

**Published:** 2017-02-15

**Authors:** Lucila Traverso, Andrés Lavore, Ivana Sierra, Victorio Palacio, Jesús Martinez-Barnetche, José Manuel Latorre-Estivalis, Gaston Mougabure-Cueto, Flavio Francini, Marcelo G. Lorenzo, Mario Henry Rodríguez, Sheila Ons, Rolando V. Rivera-Pomar

**Affiliations:** 1 Laboratorio de Neurobiología de Insectos, Centro Regional de Estudios Genómicos, Facultad de Ciencias Exactas, Universidad Nacional de La Plata, La Plata, Argentina; 2 Centro de Bioinvestigaciones (CeBio) and Centro de Investigación y Transferencia del Noroeste de Buenos Aires (CITNOBA-CONICET), Universidad Nacional del Noroeste de la Provincia de Buenos Aires, Pergamino, Argentina; 3 Instituto Nacional de Salud Pública, Cuernavaca, México; 4 Centro de Referencia de Vectores (CeReVe), Dirección de Enfermedades Transmisibles por Vectores, Ministerio de Salud de la Nación Argentina, Santa María de Punilla, Córdoba, Argentina; 5 Centro de Endocrinología Aplicada y Experimental, Facultad de Medicina, Universidad Nacional de La Plata, La Plata, Buenos Aires, Argentina; 6 Grupo de Comportamento de Vetores e Interação com Patógenos-CNPq, Centro de Pesquisas René Rachou–FIOCRUZ, Belo Horizonte, Brazil; Liverpool School of Tropical Medicine, UNITED KINGDOM

## Abstract

**Background:**

Triatomine insects are vectors of *Trypanosoma cruzi***,** a protozoan parasite that is the causative agent of Chagas’ disease. This is a neglected disease affecting approximately 8 million people in Latin America. The existence of diverse pyrethroid resistant populations of at least two species demonstrates the potential of triatomines to develop high levels of insecticide resistance. Therefore, the incorporation of strategies for resistance management is a main concern for vector control programs. Three enzymatic superfamilies are thought to mediate xenobiotic detoxification and resistance: Glutathione Transferases (GSTs), Cytochromes P450 (CYPs) and Carboxyl/Cholinesterases (CCEs). Improving our knowledge of key triatomine detoxification enzymes will strengthen our understanding of insecticide resistance processes in vectors of Chagas’ disease.

**Methods and findings:**

The discovery and description of detoxification gene superfamilies in normalized transcriptomes of three triatomine species: *Triatoma dimidiata*, *Triatoma infestans* and *Triatoma pallidipennis* is presented. Furthermore, a comparative analysis of these superfamilies among the triatomine transcriptomes and the genome of *Rhodnius prolixus*, also a triatomine vector of Chagas’ disease, and other well-studied insect genomes was performed. The expression pattern of detoxification genes in *R*. *prolixus* transcriptomes from key organs was analyzed. The comparisons reveal gene expansions in Sigma class GSTs, CYP3 in CYP superfamily and clade E in CCE superfamily. Moreover, several CYP families identified in these triatomines have not yet been described in other insects. Conversely, several groups of insecticide resistance related enzymes within each enzyme superfamily are reduced or lacking in triatomines. Furthermore, our qRT-PCR results showed an increase in the expression of a CYP4 gene in a *T*. *infestans* population resistant to pyrethroids. These results could point to an involvement of metabolic detoxification mechanisms on the high levels of pyrethroid resistance detected in triatomines from the Gran Chaco ecoregion.

**Conclusions and significance:**

Our results help to elucidate the potential insecticide resistance mechanisms in vectors of Chagas’ disease and provide new relevant information for this field. This study shows that metabolic resistance might be a contributing cause of the high pyrethroid resistance observed in wild *T*. *infestans* populations from the Gran Chaco ecoregion, area in which although subjected to intense pyrethroid treatments, vector control has failed. This study opens new avenues for further functional studies on triatomine detoxification mechanisms.

## Author summary

One of the principal goals of the last decades regarding public health in Latin America was the reduction of the geographic range and infestation rates of triatomines, the insect vectors of Chagas’ disease. However, the elimination of vector transmission has failed in the Gran Chaco ecoregion, even in areas subjected to intense vector control efforts with pyrethroid insecticides. Therefore, detecting the reasons for the persistence of these vectors has been recognized as a priority by public health authorities. Metabolic insecticide resistance due to enhancement of detoxification activity is one of the principal resistance mechanisms observed in insects. In this study, we analyzed the main enzyme superfamilies known to be involved in insecticide resistance in other insects in four important Chagas’ disease vector species from different regions of Latin America. This was made in a comparative manner using well-studied insect genomes as references. We have found gene expansions for several families related to insecticide resistance, while others showed a reduction. Augmented expression of a CYP gene may suggest contribution to pyrethroid resistance in highly resistant populations of *Triatoma infestans* from the Gran Chaco. Our results increase the knowledge of potential insecticide resistance mechanisms in vectors of Chagas’ disease and provide relevant information to this field.

## Introduction

Triatomines are hematophagous insects of the order Hemiptera, family Reduviidae, which are widely distributed throughout the Americas. This insect subfamily includes the vectors of *Trypanosoma cruzi*, the causative agent of Chagas' disease affecting around 8 million people in Central and South America [1]. The most important vector species are *Triatoma infestans* in the South Cone (especially in Argentina and Bolivia) and *Triatoma dimidiata* in Northern South America (Colombia, Venezuela, Ecuador and Peru), Central America and Mexico. *Rhodnius prolixus* (also present in those countries of the North of South America and Central America) and *Triatoma pallidipennis* in Mexico can also transmit *T*. *cruzi* [1]. Because there is still no available vaccine or effective treatment for the chronic stage of the disease, vector control remains the main method to reduce the risk of infection.

Vector control efforts have been based mainly on the application of pyrethroid insecticides, achieving an important reduction in the geographic range and level of triatomine infestation of human dwellings [2–4]. However, in the Gran Chaco region, a 1.1 million km^2^ ecoregion covering part of Argentina, Bolivia and Paraguay, the elimination of triatomines failed despite intensive use of pyrethroids [5,6]. Resistance to pyrethroids seems to be an important factor for the persistence of vectorial transmission of Chagas' disease in the Gran Chaco [5]. The detection of resistant populations of *T*. *infestans* confirmed the potential of triatomines to develop high levels of resistance (with observed resistance ratios from 35 to >1000 when compared with a sensitive strain [6]), contradicting initial assumptions [7,8]. The main cause of the extremely high levels of resistance in *T*. *infestans* seems to be the appearance of *kdr* mutations affecting the target site of pyrethroids, the voltage-gated sodium channel [6,9–11]. We have observed that *T*. *infestans* resistant populations having the same *kdr* mutation at similar frequencies present very different resistance ratios [6]. These data suggest that other mechanisms could be involved in triatomine insecticide resistance. Indeed, metabolic resistance [12,13] and a decreased cuticular penetration [14] have already been reported in particular *T*. *infestans* resistant populations, supporting this hypothesis.

In insects, detoxification mechanisms transform toxins to non-toxic molecules through Phase I and Phase II reactions. Phase I includes oxidation, reduction and hydrolysis, whereas Phase II occurs through enzyme mediated conjugated reactions to convert the primary products of Phase I into water soluble derivatives, which can be excreted [15]. Genes encoding detoxification enzymes include members of Phase I (Cytochromes P450-CYPs and Carboxyl/Cholinesterases-CCEs), and Phase II (Glutathione Transferases-GSTs) categories, forming broad gene superfamilies. Specific groups of genes within these superfamilies can mediate metabolic resistance to insecticides by means of alterations in their activity [16–18]. Besides their role in detoxification, members of GST, CYP and CCE superfamilies are involved in biosynthesis of endogenous compounds and degradation of odor molecules [19,20].

Here we present a comprehensive study of triatomine detoxification gene superfamilies, discovered in normalized transcriptomes of three epidemiologically relevant triatomine vector species: *T*. *infestans*, *T*. *dimidiata* and *T*. *pallidipennis*. Additionally, we have data-mined the *R*. *prolixus* genomic sequences to identify detoxification-related superfamilies and have characterized the expression of these superfamilies in different tissue-specific transcriptomes from *R*. *prolixus*. As insect antennae are crucial organs in the detection and processing of olfactory stimuli, we analyzed the expression patterns of GSTs, CYPs and CCEs in three antennal transcriptomes from 5^th^ instar larvae, male and female adults of *R*. *prolixu*s, with the objective of identifying potential odor/pheromone degrading enzymes. The dramatic physiological alterations triggered by blood feeding in triatomines are mostly regulated by the central nervous system (CNS) [21–23], which is also the target of all the neurotoxic insecticides used. Hence, we studied the changes in expression patterns observed for detoxification gene superfamilies in the CNS of male and female adults of *R*. *prolixus* at four different feeding conditions (unfed, 1, 4 and 24 hours post blood meal). Finally, we analyzed *R*. *prolixus* tissue-specific libraries from anterior and posterior midgut, hindgut [24], Malpighian tubules, fat body, gonads and whole body to obtain transcriptomic information of the detoxification gene superfamilies in those tissues. These libraries were constructed by Oliveira *et al*. and their sequencing output is publicly available at http://rhodnius.iq.ufrj.br.

The results presented here give a comprehensive panorama of triatomine detoxification genes and their expression in different tissues and under different physiological conditions, constituting an important platform for further physiological hypotheses and experimentation.

## Methods

### Ethics statement

Pigeons used in this study were housed, cared, fed and handled in accordance with resolution 1047/2005 (National Council of Scientific and Technical Research, CONICET) regarding the national reference ethical framework for biomedical research with laboratory, farm, and nature collected animals (Marco Ético de Referencia para las Investigaciones Biomédicas en Animales de Laboratorio, de Granja y Obtenidos de la Naturaleza), which is in accordance with the standard procedures of the Office for Laboratory Animal Welfare, Department of Health and Human Services, NIH and the recommendations established by the 2010/63/EU Directive of the European Parliament, related to the protection of animals used for scientific purposes. Biosecurity considerations are in agreement with CONICET resolution 1619/2008, which is in accordance with the WHO Biosecurity Handbook (ISBN 92 4 354 6503). The collection of insects in dwellings was performed in agreement with the Argentinean National Health Ministry ethic requirements.

### Data availability statement

The Roche 454 reads for *T*. *dimidiata*, *T*. *infestans* and *T*. *pallidipennis* were submitted to NCBI Sequence Read Archives (SRA) under the Bioproject ID number PRJNA304741 (SRA numbers: SAMN04317638 for *T*. *infestans*; SAMN04317639 for *T*. *dimidiata* and SAMN04317640 for *T*. *pallidipennis*). For reasons of legibility, we assigned an abbreviated name for each sequence in our manuscript. The correspondence between contig and assigned name is presented in Supplementary S1 File. All *Triatoma* spp. protein sequences of contigs used to construct the phylogenetic trees are available in Supplementary S2 File.

### Insecticide resistance gene identification and structural analysis

In order to identify genes involved in insecticide resistance we used as queries the PFAM domains [25] of GSTs (PF02798 and PF00043), CYPs (PF00067) and CCEs (PF00135) to perform BLAST searches [26] on the predicted transcriptome of *R*. *prolixus* (RproC3.1 version; available at www.vectorbase.org) and the transcriptomes of *T*. *infestans*, *T*. *dimidiata* and *T*. *pallidipennis* (Martínez-Barnetche *et al*., manuscript in preparation). The microsomal GSTs of *Triatoma* spp. were identified using the orthologues from *R*. *prolixus* [27], *Drosophila melanogaster*, *Anopheles gambiae*, *Apis mellifera* and *Acyrthosiphon pisum*.

Briefly, we generated normalized libraries with the kit Mint-2 (Evrogen, Moscow, Russia) using RNA extracted from eggs, unfed and fed insects from larvae (1^st^ to 5^th^ instar) and adults (males and females) of *T*. *infestans*, *T*. *dimidiata* and *T*. *pallidipennis*. Libraries were sequenced on the platform GS FLX+ (454-ROCHE, Basel, Switzerland) and the quality-trimmed reads were subjected to *de novo* assembly with the GS DeNovo assembler v.2.8 software in cDNA mode, using the default parameters. Completeness assessment of the transcriptomes for *Triatoma* spp. was evaluated searching the Core Eukaryotic Genome Dataset (CEGMA)[28] and the Benchmarking Universal Single Copy Orthologues (BUSCO) [29]. The estimated coverages were between 85.8% (BUSCO) and 94.3% (CEGMA) for *T*. *dimidiata*; 82.2% (BUSCO) and 90.6% (CEGMA) for *T*. *infestans* and 86.9% (CEGMA) and 95.2% (BUSCO) for *T*. *pallidipennis*. Following the same approach, the completeness of the *R*. *prolixus* genome was between 97.0% (BUSCO) and 97.8% (CEGMA).

After BLAST searches, the non-redundant resulting sequences were used to perform an InterProScan search [30] using the Gene3d, PfamA and SuperFamily applications to obtain the protein sequences of interest. After this step, additional tBLASTn searches on the National Center for Biotechnology Information (NCBI) non redundant (nr) database were performed to assess for false positives. In those cases where two or more proteins were obtained from one transcript due to insertions or deletions during sequencing, they were manually reconstructed into a single transcript using as references homologue protein sequences from other insects. Prediction of the subcellular localization for each protein was performed with EuK-mPLoc 2.0 [31–33] (Supplementary S1 File). The number and location of the transmembrane domains were predicted using the online server TMHMM Server v. 2.0 [34] (Supplementary S1 File). The protein motif search was performed online using MOTIF (http://www.genome.jp/tools/motif/) (Supplementary S1 File).

### Phylogenetic analysis

We compared protein sequences related to detoxification superfamilies identified in the *R*. *prolixus* genome and *Triatoma* spp. transcriptomes with orthologues previously published for *D*. *melanogaster* and other insect species [18,35–41]. Phylogenies for each protein family were based on sequence alignments generated by CLUSTAL Ω [42] using the corresponding Hidden Markov Model for each PFAM superfamily domain as an external profile. Phylogenetic trees were inferred with the software BEAST v1.8.3 [43] in the CIPRES Science Gateway [44]. Beauti v1.8.3 [43] was used to generate the BEAST input files. Two independent chains were run with the same settings until convergence was achieved (effective sample size values >200), as calculated in the program Tracer v1.6.0 (http://beast.bio.ed.ac.uk/Tracer). To achieve convergence, the number of generations used for each run varied between 5 to 30 million, depending on the tree. Each pair of runs was combined using LogCombiner v1.8.3 [43] discarding the first 10% of each chain as a burn-in. The maximum clade credibility tree was generated using TreeAnnotator v1.8.3 [43]. The results were visualized with iTol online tool [45]. To maintain the legibility of the figures, the phylogenetic analysis included only *D*. *melanogaster* as a reference, and the analysis including more insect species is presented as Supplementary S4 File.

### Mapping reads

Filtered and trimmed Illumina reads for the *R*. *prolixus* CNS libraries were obtained from Ons *et al*. 2016 [46]. Subsequently, these reads and *R*. *prolixus* genome sequences (assembly version RproC3.13) were mapped to the annotated genome assembly by means of TopHat v. 2.0.11 [47]. Using mapped read information, a transcriptome assembly and the corresponding gene expression levels (expressed as Fragments Per Kilobase of transcript per Million mapped reads or FPKM) were obtained for each library using Cufflinks v. 2.1.0 [47]. Cuffmerge [47] was used for joining the predicted transcriptome assemblies and the annotated genome GTF file. Finally, Cuffdiff [47] allowed comparing FPKM values for each annotated transcript in the different conditions. In the case of *R*. *prolixus* antennae, FPKM values were obtained from Latorre-Estivalis *et al*. 2017 [48].

### Transcript abundance and tissue expression analysis

Heat maps showing gene expression (presented as Log10 FPKM +1) of the target genes in the CNS from pooled male and female *R*. *prolixus* adult insects at different times post-blood meal (unfed as basal condition, 1, 4 and 24 hours post-feeding) and in the antennae (including 5^th^ instar larvae, female and male adult libraries) were prepared using R package ggplot2 (www.ggplo2.org).

For tissue-specific expression analysis, we used public transcriptomes from anterior midgut, fat body, Malpighian tubules, posterior midgut, ovary, rectum, testes and whole body (see [24] for a detailed description on the construction of these transcriptomes). We performed local tBLASTn [26] to correlate the assembled contigs from those transcriptomes to an ID number in v3.1 of *R*. *prolixus* gene set. For this, we used a database containing all the assembled contigs (http://rhodnius.iq.ufrj.br), and the sequences of detoxification superfamilies of *R*. *prolixus* as queries. We analyzed the New-S1-Full.xlsx spreadsheet (available at http://rhodnius.iq.ufrj.br/), to determine the number of reads for every detoxification-related transcript in each tissue. This analysis allowed the estimation of the tissue-specific expression pattern for each transcript and helped to obtain insights about their relative expression comparing different structures.

### Expression analysis in resistant populations

**Insects**—Field insects of *T*. *infestans* were collected from infested houses in El Juramento, an Argentinean population with a resistance ratio higher than 2,000 and with presence of the L925I *kdr* mutation. Sensitive laboratory population of *T*. *infestans* (without exposure to insecticides for more than 30 generations) from the Reference Center for Vectors of the Ministry of Public Health of Argentina was used as a reference. The two populations analyzed are sensitive to fenitrothion (for a detailed description on sensitive and resistant strains used see [6]).

**qRT-PCR**—Unfed 5^th^ instar larvae of the reference and resistant *T*. *infestans* strains were used for transcript abundance comparisons. From each insect, complete CNS, anterior and posterior midgut, rectum, Malpighian tubules and fat body from the abdomen (separated from tracheae and other tissues) were dissected and pooled in a microtube containing Trizol (Ambion, Sao Paulo, Brazil); this reagent was also used for total RNA extraction, according to the manufacturer's instructions. A total of 1 μg of RNA was treated with DNAseI (Promega, Wisconsin, USA) and used to synthesize cDNA by means of M-MLV Reverse Transcriptase kit (Promega). cDNA amplifications of *T*. *infestans* were performed on triplicate in a 25 μL final volume (primers detailed in Supplementary S3 File). α-Tubulin, β-Actin and G6PDH were used as housekeeping genes; they were previously reported as stable genes for qRT-PCR experiments in *R*. *prolixus* [49]. The program used in the amplifying reaction was 95°C for 5 min, and 39 cycles of 95°C for 30 sec; 58°C for 30 sec and 72°C for 30 sec in the Mini Opticon Real-Time PCR Detector Separate MJR (Bio-Rad Laboratories, California, USA). Real-time data were collected through the CFX Manager 3.0 software (Bio-Rad Laboratories). Results were statistically analyzed using One-Way ANOVA.

## Results and discussion

The GST, CYP and CCE protein superfamilies have been involved in biosynthetic and signaling processes and some members of these superfamilies have been directly implicated in metabolic resistance to insecticides [16–18]. We compared the repertories of detoxification gene superfamilies in normalized transcriptomes of *T*. *infestans*, *T*. *dimidiata* and *T*. *pallidipennis*. Furthermore, we analyzed the corresponding enzymes in the genome of *R*. *prolixus* (most of them annotated in a recent paper [27]) and those of other well-studied insects: two Dipterans (*D*. *melanogaster* and *An*. *gambiae*), one Hymenoptera (*A*. *mellifera*) and one Hemiptera (*A*. *pisum*). Sequences from *D*. *melanogaster* and *R*. *prolixus* were used to classify *Triatoma* spp. detoxification genes repertories. Finally, the expression patterns of detoxification genes were analyzed in *R*. *prolixus* transcriptomes from different tissues.

Complete genomes of triatomines, except for *R*. *prolixus*, are not yet available, being normalized transcriptomes an alternative approach for obtaining a broad catalog of transcripts. Even considering the normalization process performed during our experimental design, we cannot rule out failures in the detection of some transcripts with low expression levels or restricted expression patterns. Nevertheless, in previous studies, we were able to detect many low expressed transcripts in our normalized transcriptomes, such as embryonic developmental genes or neuropeptide precursors [50,51]. In addition, most of the gene superfamily members analyzed here are involved in different physiological processes and are highly expressed in several insect tissues [20], facilitating their detection in normalized transcriptomes.

Our completeness estimations suggest that the transcriptomic datasets presented here are complete enough for the global characterization of detoxification gene families and inter-species comparisons. We present several genome to transcriptome comparisons between *R*. *prolixus* and *Triatoma* spp. However, these comparisons should be cautiously considered due to the technical limitations and certain degree of underrepresentation in our transcriptomes.

### Cytochromes P450

**Fig 1:**
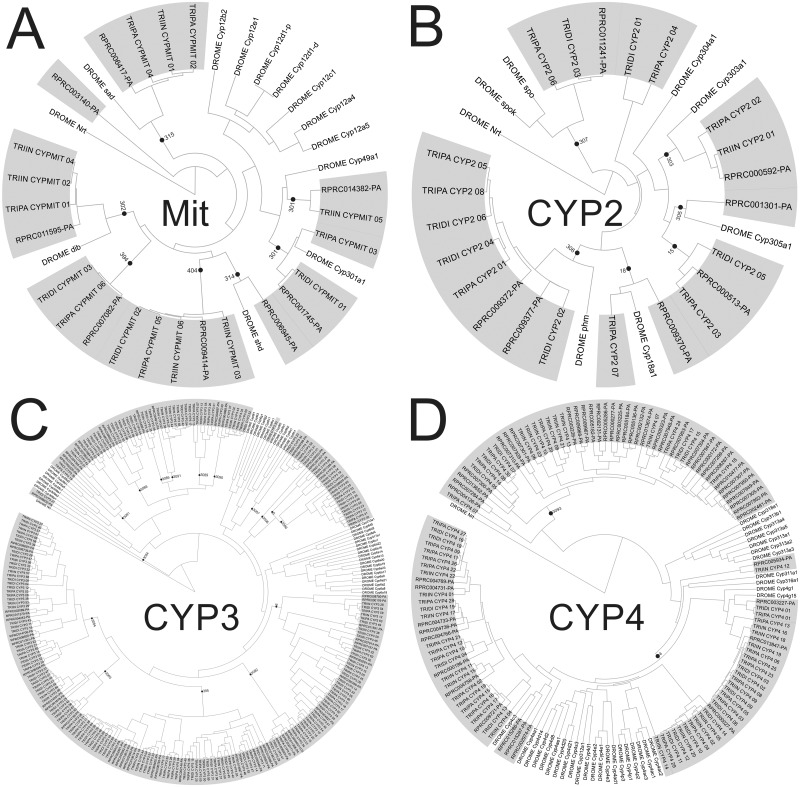
Phylogeny of the CYP superfamily from *R*. *prolixus* (VectorBase ID shown), *T*. *infestans* (TRIIN), *T*. *dimidiata* (TRIDI), *T*. *pallidipennis* (TRIPA) and *D*. *melanogaster* (DROME). (A) Phylogeny of mitochondrial clade. (B) Phylogeny of CYP2 clade. (C) Phylogeny of CYP3 clade. (D) Phylogeny of CYP4 clade. The sequence of Neurotactin from *D*. *melanogaster* (CG9704) was used as outgroup. The triatomine sequences are painted in grey.

CYPs are critically involved in metabolic processes such as the biosynthetic pathways of endogenous signaling molecules (e.g., 20-hydroxyecdysone and juvenile hormone), the degradation of pheromones and the hydroxylation of fatty acids [19]. They are also critically implicated in the detoxification response to xenobiotics, including synthetic insecticides, and are fundamental factors in the adaptation of insects to chemical stress. Insect CYP genes fall into four major clades: CYP2, CYP3, CYP4 and mitochondrial clade. Each one of these clades are further subdivided in several families and subfamilies [19]. New families belonging to clades CYP3 and CYP4 were recently identified in *R*. *prolixus* [27]. Our analysis confirmed that, with exception of CYP3096, these new families are also present in other triatomine species (Table 1; Fig 1 and Supplementary S4 File). We detected a total of 87 CYP transcripts in *T*. *dimidiata*, 94 in *T*. *infestans*, 113 in *T*. *pallidipennis* and 119 in *R*. *prolixus*, these numbers being higher than those found in *A*. *pisum* (64), *D*. *melanogaster* (88), and *A*. *mellifera* (46) (Table 1).

**Table 1:** Gene numbers of GST, CYP and CCE superfamilies from *R*. *prolixus* genome and from *T*. *dimidiata*, *T*. *infestans* and *T*. *pallidipennis* transcriptomes in comparison with other insect species. Numbers were derived from Claudianos *et al*. (2006), Feyereisen *et al*. (2006 and 2012), Oakeshott *et al*. (2010), Ramsey *et al*. (2010), Shi *et al*. (2012) and http://drnelson.uthsc.edu/aphid.htm. (*) Shi *et al*. (2012) found one Epsilon GST in *A*. *pisum* while Ramsey *et al*. (2010) did not find any Epsilon GST.

Superfamily	Class/Clade	*R*. *prolixus*	*T*. *dimidiata*	*T*. *infestans*	*T*. *pallidipennis*	*D*. *melanogaster*	*An*. *gambiae*	*A*. *mellifera*	*A*. *pisum*
**CYPs**	**Mitochondrial**	**8**	**3**	**6**	**6**	**11**	**9**	**6**	**8**
	Families	*CYP301*, *302*, *314*,	*CYP301*,	*CYP301*, *302*,	*CYP301*, *302*,	*CYP12*,*49*,*301*,	*CYP12*,*49*,*301*,	*CYP301*, *302*,	*CYP301*, *302*,
		*315*, *394*, *404*	*394*, *404*	*315*, *404*	*315*, *394*, *404*	*302*, *314*,*315*	*302*, *314*,*315*	*314*, *315*, *334*	*314*, *315*, *353*
	**CYP2**	**7**	**6**	**1**	**8**	**7**	**10**	**8**	**10**
	Families	*CYP15*, *18*, *303*, *305*	*CYP15*, *306*, *307*	*CYP303*	*CYP15*, *18*, *303*	*CYP18*, *303–307*	*CYP15*, *303–307*	*CYP15*, *18*, *303*,	*CYP15*, *18*, *303*,
		*306*, *307*			*306*, *307*			*305–307*, *342*, *343*	*305–307*
	**CYP3**	**55**	**59**	**65**	**69**	**36**	**40**	**28**	**23**
	Families	*CYP6*, *395*,	*CYP6*, *395*,	*CYP6*, *395*,	*CYP6*, *395*,	*CYP6*, *9*, *28*,	*CYP6*, *9*, *329*	*CYP6*, *9*, *336*	*CYP6*
		*3084–3092*, *3096*	*3084–3092*	*3084–3091*	*3084–3092*	*308–310*, *317*			
	**CYP4**	**49**	**19**	**22**	**30**	**32**	**46**	**4**	**23**
	Families	*CYP4*, *3093*	*CYP4*, *3093*	*CYP4*, *3093*	*CYP4*, *3093*	*CYP4*, *311–313*,	*CYP4*, *325*	*CYP4*	*CYP4*, *380*
						*316*, *318*			
	**Subtotal**	**119**	**87**	**94**	**113**	**88**	**105**	**46**	**64**
**CCEs**	**Dietary class**								
	A clade	0	0	0	0	0	0	5	5
	B clade	0	0	0	0	13	16	3	0
	C clade	0	0	0	0	0	0	0	0
	**Pheromone/hormone processing class**								
	D clade	0	0	0	0	3	0	1	0
	E clade	40	25	18	17	2	4	2	18
	F clade	0	0	0	0	3	6	2	0
	G clade	0	0	0	0	0	4	0	0
	**Neuro/developmental class**								
	H clade	2	1	0	1	5	10	1	0
	I clade	1	0	0	0	1	1	1	1
	J clade	2	0	1	0	1	2	2	2
	K clade	1	0	0	0	1	1	1	1
	L clade	13	1	0	0	4	5	5	3
	M clade	2	0	0	0	2	2	1	0
	**Subtotal**	**61**	**27**	**19**	**18**	**35**	**51**	**24**	**30**
**GSTs**	Delta	1	1	1	1	11	17	2	16
	Epsilon	0	0	0	0	14	8	0	1 (0) *
	Omega	1	2	0	1	4	1	2	2
	Sigma	7	13	9	5	1	1	4	6
	Theta	3	2	2	1	4	2	1	2
	Zeta	1	1	0	1	2	1	1	0
	Microsomal	1	3	2	2	3	3	2	2
	Unknown	0	0	0	0	1	2	1	3
	**Subtotal**	**14**	**22**	**14**	**11**	**40**	**35**	**13**	**32**
**Total**		**194**	**136**	**127**	**142**	**163**	**191**	**83**	**126**

#### The mitochondrial and CYP2 clades

**Fig 2:**
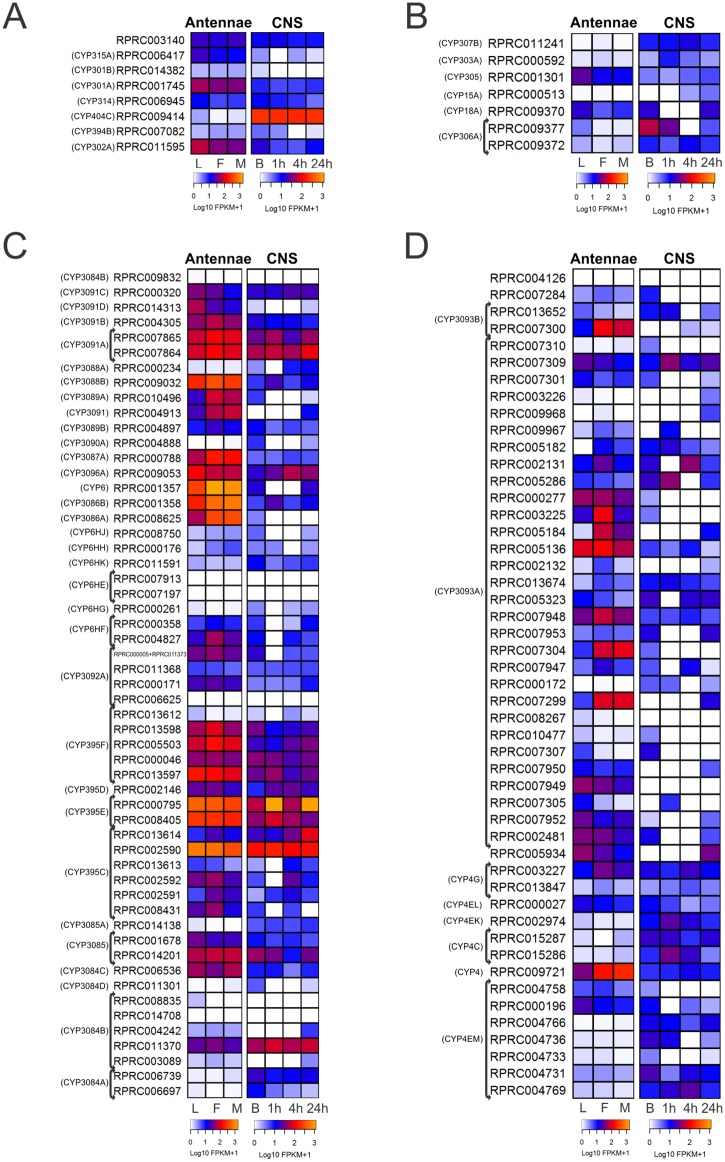
Heat maps comparing expression levels of CYP members in antennae and the central nervous system (CNS) of *R*. *prolixus* in different conditions. (A) Mitochondrial clade. (B) CYP2 clade. (C) CYP3 clade. (D) CYP4 clade. In each figure, on the left, expression levels in larvae (L), female (F) and male (M) adult antennae; on the right, expression levels in the central nervous system (CNS) from adult bugs in basal condition (B), one, four and twenty-four hours after blood ingestion. Expression levels (represented as Log10 FPKM +1) were depicted with a color scale, in which white represents lower expression and yellow represents higher expression. The phylogenetic classification of CYP members according to Schama *et al*. (2015) is shown on the left.

Mitochondrial CYP genes are absent in plants or fungi, and seem to be restricted to animals. Some mitochondrial CYP genes are involved in essential physiological functions, and are conserved among insect species. This is the case of the *Halloween* genes: 302A or *disembodied (dib)*, 314A or *shadow (shd)* and 315A or *sad (sad)*, which are involved in ecdysteroid biosynthesis [19]. In triatomine databases, the mitochondrial clade was represented by 8 genes in *R*. *prolixus*, 6 in *T*. *infestans*, 6 in *T*. *pallidipennis* and 3 in *T*. *dimidiata* (Fig 1A, Table 1 and Supplementary S4 File). The orthologues of *Dmel-sad*, *Dmel-dib*, *Dmel-shd* and *Dm-301a1* were identified in triatomines (Fig 1A). None of the triatomine transcripts identified were grouped into the mitochondrial CYP12 family (Fig 1A), which has been related to insecticide resistance [19]. This family is also absent in *A*. *pisum* and *A*. *mellifera* [39,40] and would reflect a lack of detoxifying capability in the mitochondrial clade. We found that *Rprodib* (RPRC011595) and the orthologue of *Dm-301a1* (RPRC001745) were the most expressed transcripts in *R*. *prolixus* antennae (Fig 2A). In the *R*. *prolixus* CNS, only RPRC009414 presented a high expression (family 404C, Fig 2A). This was also the most expressed mitochondrial CYP gene in the *R*. *prolixus* transcriptomic databases from Oliveira *et al*. (Supplementary S5 File).

Some CYP2 members are highly conserved among insects. Some orthologues of the *Halloween* genes 307A (*Spook/Spookier*) and 306A (*Phantom*), involved in ecdysteroid biosynthesis [52–54], were identified (Fig 1B). The orthologues of the *Dm-18a1* gene were identified in *R*. *prolixus* (RPRC009370) and *T*. *pallidipennis* (TRIPA_CYP2_07) (Fig 1B). Finally, the CYP15 family was represented by the transcripts RPRC000513, TRIDI_CYP2_05 and TRIPA_CYP2_03, the CYP305 family was represented by RPRC001301, and CYP303 family was represented by RPRC000592, TRIIN_CYP2_01 and TRIPA_CYP2_02.

The expression of most CYP2 genes in the *R*. *prolixus* CNS and antennal transcriptomes (Fig 2B) and the transcriptomic databases from Oliveira *et al*. (Supplementary S5 File) was low. The orthologues of *Dm-18a1* and *Dm-305a1* were the most expressed transcripts in antennae, especially in larvae. The expression of RPRC009377, phylogenetically related to *Dm-phm*, seems to decrease after blood ingestion in the CNS. The expression of CYP18A and CYP15A transcripts in the CNS remarkably changes over the different times post-blood meal (Fig 2B). The involvement of these genes in the detoxification processes that are triggered by blood feeding remains to be determined in triatomines.

#### The CYP3 clade: Triatomine-specific families and the absence of CYP9

CYP3 is the more abundant CYP clade in insects and its members are usually organized in large clusters in insect genomes. Genes from this clade belonging to CYP6 and CYP9 families have been involved in xenobiotic metabolism and insecticide resistance [19]. In *R*. *prolixus*, several CYP members were previously identified in transcriptomes from different tissues and whole body [24]. These transcripts were highly expressed in all segments of the gut (anterior midgut, posterior midgut and rectum) when compared with whole body [24], possibly reflecting another function of CYPs related to the metabolism of steroids and lipids.

A total of 10 new CYP3 families have been recently described in *R*. *prolixus* genome, including CYP3084 to CYP3092 and CYP3096 [27]. CYP3 clade was the most numerous clade in triatomines (55 genes in *R*. *prolixus*, 65 in *T*. *infestans*, 59 in *T*. *dimidiata* and 69 in *T*. *pallidipennis*), representing an expansion in relation to *D*. *melanogaster* (36), *An*. *gambiae* (40), *A*. *mellifera* (28) and *A*. *pisum* (23). Structural analysis of these expanded clades, including transmembrane domains and subcellular localization predictions are presented in Supplementary S1 File.

Our analysis reveals that all members of triatomines’ CYP3 clade were grouped into families 6, 3084, 3085, 3086, 3087, 3088, 3089, 3090, 3091, 3092, 3096 and 395 (Fig 1C). The three more represented CYP families in triatomines were: CYP395 (14 transcripts in *R*. *prolixus*, 10 in *T*. *dimidiata*, 10 in *T*. *infestans* and 22 in *T*. *pallidipennis*), CYP6 (8 transcripts in *R*. *prolixus*, 9 in *T*. *dimidiata*, 15 in *T*. *infestans* and 9 in *T*. *pallidipennis*) and CYP3084 (10 transcripts in *R*. *prolixus*, 13 in *T*. *dimidiata*, 10 in *T*. *infestans* and 10 in *T*. *pallidipennis*) (Fig 1C). A few sequences were not clearly classified in a CYP3 family (Fig 1C); all of them seem to be fragmented transcripts. A remarkable finding of this work is that the CYP9 family was not found in triatomines (Fig 1C), despite it being a large family in several insect genomes [36]. Our results suggest that this family is absent or less represented in triatomines. Hence, CYP6 is the only family within the triatomines’ CYP3 clade that has been previously involved with insecticide resistance [36].

It has been proposed that CYP duplication events could facilitate the recruitment of new members for a specialized physiological function [17]. In the case of *R*. *prolixus*, 32 CYP3 members (belonging to family 395 and the recently found 3084, 3085, 3086, 3087, 3089, 3090, 3091 and 3092 families) were grouped in seven clusters (at least three genes in the same supercontig), considering their location in the genome. The supercontigs KQ035657, KQ034396, KQ034255 and KQ034131 contain 3 CYP3 genes each. The supercontig KQ034742 contains 5 genes whereas KQ034121 contains 6 CYP3 genes. The biggest cluster within this clade was found in the supercontig KQ034111, which contains 9 genes, all of them belonging to the CYP395 family. This cluster organization and the sequence similarity among *R*. *prolixus* CYP3 transcripts suggests that they could be products of gene duplication events and would probably be involved in similar physiological processes. This clusterization has been observed in particular CYPs involved in insecticide detoxification and resistance in *An*. *gambiae* [55], *Musca domestica* [56], *D*. *melanogaster* [57] and *Helicoverpa zea* [58]. Therefore, the phylogenetic analysis and the clustered genome organization suggest a possible involvement of those CYP3 genes in triatomine insecticide resistance.

Members of CYP395, CYP3086, CYP3087, CYP3088, CYP3091 and CYP3096 presented high expression in antennae (Fig 2C). Some of these CYP3 genes may be involved in the processing of odors/pheromones in *R*. *prolixus* antennae, as it was observed for other CYP3 members in *D*. *melanogaster* [20] and *Spodoptera litoralis* [59]. In the CNS, the expression of RPRC007864 and RPRC007865 (CYP3091), RPRC011370 (CYP3084), RPRC014201 (CYP3085), and RPRC009053 (CYP3096) was high (Fig 2C). With few exceptions, members of CYP395 family presented high expression in antennae and the CNS (Fig 2C). Most CYP3 genes were highly expressed in anterior midgut, gonads and rectum, and less represented in Malpighian tubules, posterior midgut and fat body (Supplementary S5 File).

#### Triatomine specific family in CYP4 clade

CYP4 is very diverse in insect genomes [19], even though only family 4 in clade 4 has been related to insecticide resistance [60,61]. Other CYP4 members have different functions including biosynthesis of compounds [62] and odor processing [63,64]. A total of 49 genes in *R*. *prolixus*, 22 in *T*. *infestans*, 19 in *T*. *dimidiata* and 30 in *T*. *pallidipennis* belong to CYP4 clade (Table 1). With the exception of *A*. *mellifera*, which has 4 CYP4 members, the number of CYP4 clade genes identified in other insects genomes (32 in *D*. *melanogaster*, 46 in *An*. *gambiae*, and 23 in *A*. *pisum*) was similar to those from triatomines (Table 1).

In triatomines, CYP4 genes belong to family 4 (14 from *R*. *prolixus*, *T*. *infestans* and *T*. *dimidiata*, and 24 from *T*. *pallidipennis*) and the recently described 3093 [27] (Fig 1D). The latter seems to present an expansion in *R*. *prolixus* with 32 members, while *T*. *dimidiata* and *T*. *pallidipennis* presented 5 and *T*. *infestans* presented 7 transcripts. This is probably due to gene duplications in *R*. *prolixus*, since 22 transcripts grouped into 3 clusters: 6 genes were located in supercontig KQ034611 (RPRC007947, RPRC007948, RPRC007949, RPRC007950, RPRC007952, RPRC007953), 7 genes in supercontig KQ034366 (RPRC000277, RPRC002131, RPRC002132, RPRC005136, RPRC005182, RPRC005184, RPRC005286) and 9 genes in supercontig KQ034480 (RPRC007299, RPRC007300, RPRC007301, RPRC007304, RPRC007305, RPRC007307, RPRC007309, RPRC007310, RPRC002481). The cluster organization of the CYP3093 family could indicate a conserved function [17]. As clustered CYPs were previously associated to insecticide metabolism in insects [55–58], the potential role of this CYP4 family in *R*. *prolixus* detoxification processes deserves to be further analyzed.

The expression profile analysis of CYP4 genes indicates that some members of the CYP3093 family and the RPRC009721 transcript (from CYP4 family) seem to be highly expressed in adult antennae (Fig 2D). Several CYP4 members are also overexpressed in *D*. *melanogaster* antennae [20], and experimental data from other insects indicate an odor/pheromone degradation role for these enzymes [59,64]. Considering this functional information and the expression patterns observed in *R*. *prolixus* antennae, specific CYP4 genes could be involved in the degradation of sexual pheromones during the adult stage. The expression of most of CYP4 genes in the other transcriptomes analyzed was low or moderate (Fig 2D and Supplementary S5 File).

### Carboxyl/cholinesterases

CCEs hydrolyze carboxyl esters to alcohols and acids. They are subdivided in three major classes according to their function: i) the dietary class, containing esterases that metabolize a broad range of substrates including insecticides and xenobiotics from the diet; ii) the hormone and pheromone degrading class, including juvenile hormone esterases and pheromone degrading CCEs; and iii) the neuro/developmental group, containing proteins implicated in neuro/developmental functions as acetylcholinesterases (AChEs), neuroligins, neurotactins, gliotactins and others [18]. AChEs are the only catalytically active component of the neuro/developmental class [18,40].

**Fig 3:**
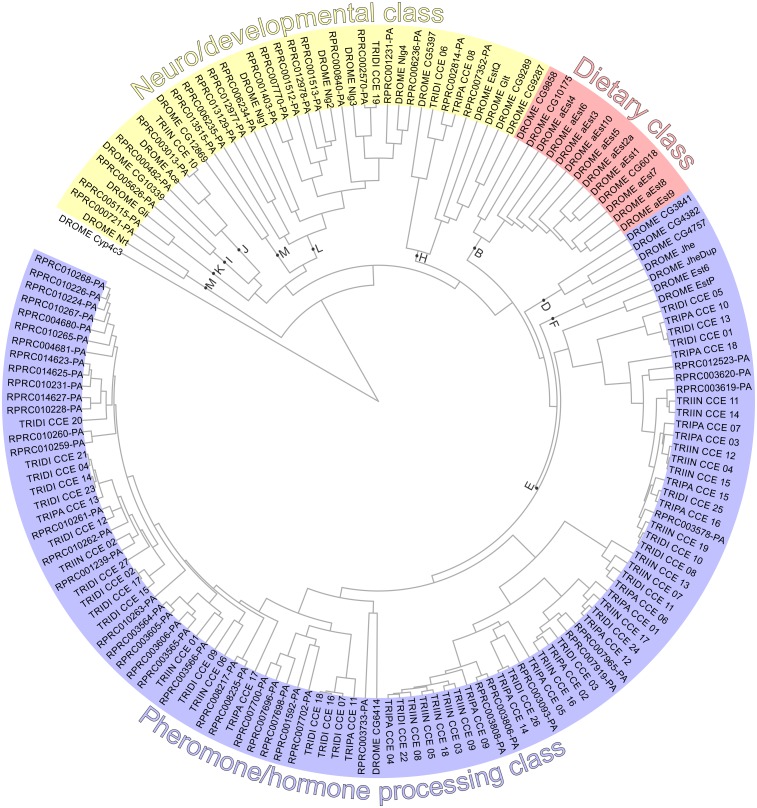
Phylogeny of the CCE superfamily from *R*. *prolixus* (VectorBase ID shown), *T*. *infestans* (TRIIN), *T*. *dimidiata* (TRIDI), *T*. *pallidipennis* (TRIPA) and *D*. *melanogaster* (DROME). The sequence of *Cyp4c3* from *D*. *melanogaster* (CG14031) was used as outgroup. The letters depicted next to the dots in the branches of the tree indicate the delimitation of each clade.

*R*. *prolixus* seems to present an expansion in the number of CCE genes (61) compared with *Triatoma* spp. (19 in *T*. *infestans*, 27 in *T*. *dimidiata* and 18 in *T*. *pallidipennis*) and the other insect genomes analyzed (35 in *D*. *melanogaster*, 51 in *An*. *gambiae*, 24 in *A*. *mellifera* and 30 in *A*. *pisum*) (Table 1; Fig 3 and Supplementary S4 File). The distribution of CCEs across clades was very different among triatomines and the other species analyzed: the dietary class is absent in triatomines, and almost all the CCEs detected are included in clade E from hormone and pheromone degrading esterases (Table 1; Fig 3 and Supplementary S4 File).

#### Dietary CCE class involved in xenobiotic metabolism is absent in triatomines

Dietary CCE class, involved in the detoxification of insecticides and xenobiotics from diet, are grouped in clades A, B and C [18,37]. An important finding of our work is the absence of dietary class CCEs in the four triatomine species analyzed here (Table 1; Fig 3 and Supplementary S4 File). Our data point to an absence or a very low representation of this class in triatomines, unlike in *A*. *pisum* which presents 5 members in this class [39]. These insects are obligate blood feeders during all their developmental stages and for this reason they are not exposed to diverse secondary metabolites from plants. This fact could explain the absence or low representation of the dietary CCE class enzymes in triatomines. The deficiency in an entire CCE class could be coupled to a lower potential for the detoxification of chemical insecticides compared to other species. However, as pyrethroid esterase activity has been demonstrated for *T*. *infestans* [12], it is possible that some esterases from the hormone and pheromone processing class may play a role in insecticide detoxification, as has been observed in other insects [18,40].

#### Hormone and pheromone processing CCEs in triatomines belong to clade E

CCEs involved in hormone and pheromone processing are distributed among the clades D, E, F and G [18,37]. As observed for *A*. *pisum* [39], triatomines showed a reduction in the diversity of this class. All the triatomines' hormone and pheromone processing CCEs (40 in *R*. *prolixus*, 18 in *T*. *infestans*, 25 in *T*. *dimidiata* and 17 in *T*. *pallidipennis*) belong to clade E. This clade is expanded in triatomines in comparison to other insect genomes (2 genes in *D*. *melanogaster* and *A*. *mellifera*, and 4 in *An*. *gambiae*) (Table 1). *R*. *prolixus* presented an expanded Clade E as a consequence of gene expansion events. A total of 29 transcripts were clustered into five supercontigs in the *R*. *prolixus* genome: KQ035483 and KQ034057 encode 3 genes each, KQ034091 and KQ034212 encode 5 genes each, and KQ034279 encodes 12 genes. The number and position of transmembrane domains, as well as the subcellular localization of this expanded clade is presented in Supplementary S1 File.

**Fig 4:**
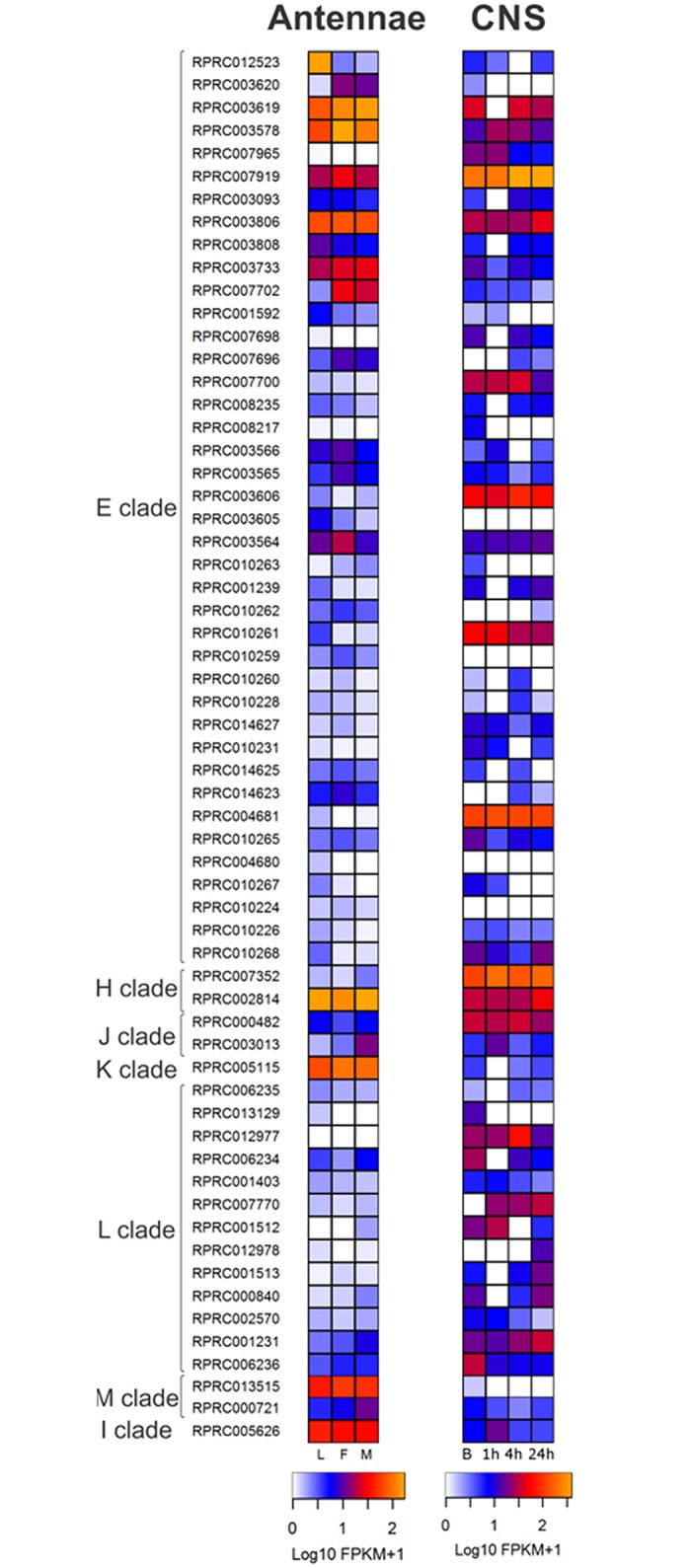
Heat maps comparing CCE expression levels in antennae and the central nervous system (CNS) of *R*. *prolixus* in different conditions. On the left, expression levels in larvae (L), female (F) and male (M) adult antennae. On the right, expression levels in central nervous system from adult bugs in basal condition (B), one, four and twenty-four hours after blood ingestion. Expression levels (represented as Log10 FPKM +1) were depicted with a color scale, in which white represents lower expression and yellow represents higher expression. The classification according the phylogenetic tree is shown on the left.

Most of Clade E members showed low expression in the *R*. *prolixus* antennal transcriptomes (Fig 4). However, RPRC003733, RPRC007919, RPRPC007702 and RPRC003806 genes, and some included in the supercontigs KQ034057 (RPRC003619 and RPRC003578) presented high expression levels in this tissue, and relatively low expression in other structures (Fig 4 and Supplementary S5 File). Considering this expression profile and that Clade E is part of the CCE pheromone/hormone processing class, some of these transcripts could be involved in odor/pheromone processing in triatomine antennae. Besides the antennae, RPRC003806 was also highly expressed in the CNS transcriptomes (Fig 4). The expression of RPRC003619, RPRC007919, RPRC007700, RPRC003606, RPRC010261 and RPRC004681 was high in the CNS transcriptomes (Fig 4).

#### Neuro/developmental CCEs

With the exception of AChE, neurodevelopmental CCEs are not catalytic, and are involved in cell-cell interactions [18]. We detected 21 transcripts of this class in the *R*. *prolixus* genome, 13 of which belong to clade L (neuroligins), 2 to clade H (glutactin), 1 to clade K (gliotactin), 1 to clade I (uncharacterized), 2 to clade M (neurotactins) and 2 to clade J (acetylcholinesterases) (Table 1). These numbers are comparable to those obtained for the other species, with a remarkable expansion of clade L (Fig 3 and Table 1). In *Triatoma* spp. transcriptomes, we identified a low number of CCEs belonging to the neurodevelopmental class: 1 clade J CCE for *T*. *infestans*; 1 clade H and 1 clade L CCE for *T*. *dimidiata*; and 1 clade H for *T*. *pallidipennis* (Fig 3). Clade I member RPRC005626, and the RPRC002814 (clade H), RPRC013515 (clade M) and RPRC005115 (clade K) genes, were highly expressed in antennae (Fig 4) and show a low expression in other tissues (Supplementary S5 File). Several genes belonging to H and L clades were highly expressed in the CNS transcriptomes (Fig 4).

### Glutathione Transferases

The GSTs are a family of enzymes widely found in eukaryotic and prokaryotic cells. These enzymes can use glutathione in reactions, contributing to the biodegradation and elimination of xenobiotics and reactive oxygen species generated during aerobic metabolism [16]. Depending on their location in the cell, the insect GSTs are classified in microsomal and cytosolic groups. Within the cytosolic group, Delta, Epsilon, Omega, Sigma, Theta and Zeta classes may be represented in insects. Among these, Delta and Epsilon classes are exclusive for insects, and are the main GST classes that have been implicated in insecticide resistance [16]. Ribeiro *et al*. [24] found 7 GST transcripts expressed in *R*. *prolixus* gut structures.

GST genes seem poorly represented in the transcriptomes of *T*. *infestans*, *T*. *dimidiata* and *T*. *pallidipennis* (14, 22 and 11 GST encoding genes, respectively), as well as in the genome of *R*. *prolixus* (14) when compared to other insect genomes (Table 1). The number of cytosolic GSTs in triatomines did not greatly differ from that found in *A*. *mellifera* (Table 1). The Dipterans and *A*. *pisum* present the highest numbers of GSTs genes (40 for *D*. *melanogaster*, 35 for *An*. *gambiae* and 32 in *A*. *pisum*) (Table 1).

**Fig 5:**
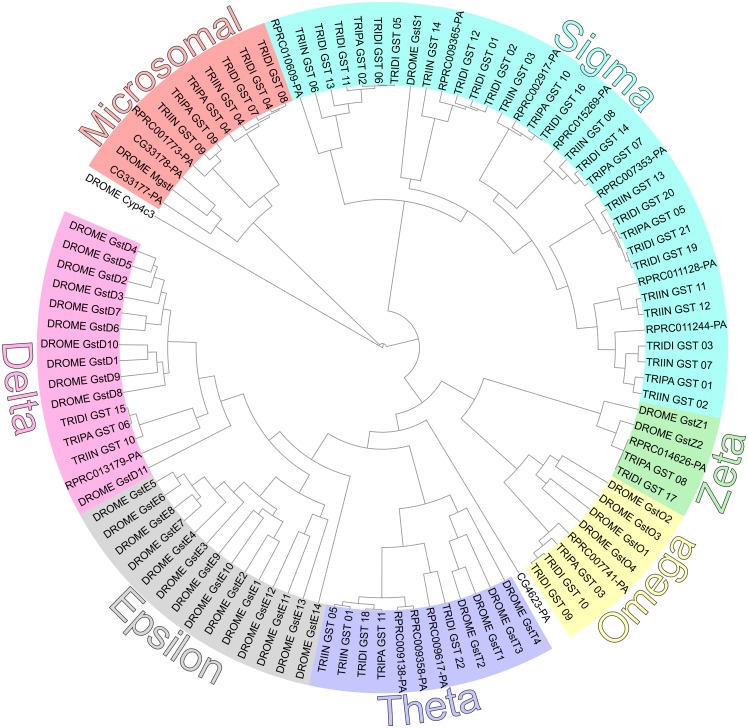
Phylogeny of the Glutathione Transferase superfamily from *R*. *prolixus* (VectorBase ID shown), *T*. *infestans* (TRIIN), *T*. *dimidiata* (TRIDI), *T*. *pallidipennis* (TRIPA) and *D*. *melanogaster* (DROME). The sequence of *Cyp4c3* from *D*. *melanogaster* (CG14031) was used as outgroup.

Microsomal GSTs have not been reported to be involved in the metabolism of insecticides, and represent a small subset of the GST complement in triatomines, with 1 representative in *R*. *prolixus*, 3 in *T*. *dimidiata*, and 2 in T. *infestans* and *T*. *pallidipennis* (Table 1 and Fig 5).

#### Delta and Epsilon GSTs

It has been suggested that GSTs, particularly those of Delta and Epsilon classes, are important in the plasticity of insects to cope with selection pressures from the environment [16]. Delta class is highly represented in the genomes of *D*. *melanogaster*, *An*. *gambiae* and *A*. *pisum* (11, 17 and 16 members, respectively). The Epsilon class is represented by 14 members in *D*. *melanogaster* and 8 in *An*. *gambiae*, while it is absent in *A*. *mellifera* and the reports of *A*. *pisum* are contradictory in the literature [35,39] (Table 1). A small subset of Delta and Epsilon classes of insect GSTs catalyzes the dehydrochlorination of the organochlorine insecticide DDT, whereas other members of these classes metabolize organophosphate insecticides [65–68]. DDT showed a low efficacy against triatomines [5]. However, we found only one member of the Delta class and no members of the Epsilon class in triatomines (Table 1 and Fig 5), suggesting that the GST activity would not be the main DDT detoxification mechanism in triatomines.

**Fig 6:**
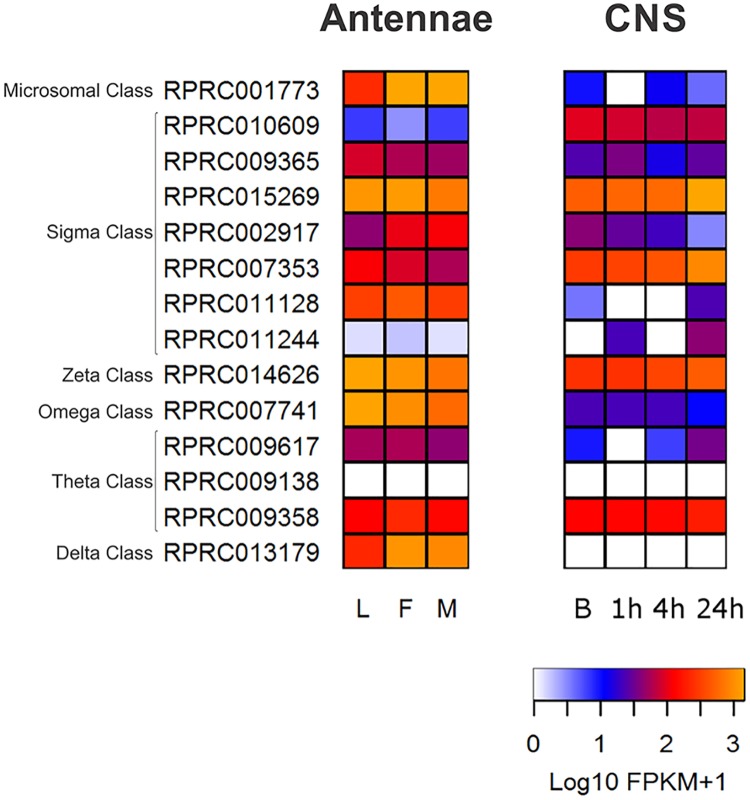
Heat maps comparing Glutathione Transferase expression levels in antennae and the central nervous system (CNS) of *R*. *prolixus* in different conditions. On the left, expression levels in larvae (L), female (F) and male (M) adult antennae. On the right, expression levels in the central nervous system from adult bugs in basal condition (B), one, four and twenty-four hours after blood ingestion. Expression levels (represented as Log10 FPKM +1) were depicted with a color scale, in which white represents lower expression and yellow represents higher expression. The classification according the phylogenetic tree is shown on the left.

The analysis of Delta GST in *R*. *prolixus* transcriptomes revealed no expression in the CNS (Fig 6), gonads, fat body, Malpighian tubules or digestive system, except for a low relative expression in the rectum (Supplementary S5 File). Delta GST was highly expressed in all antennal libraries (Fig 6), suggesting a potential role in the degradation of odor/pheromone molecules in *R*. *prolixus*, as it was observed in *Manduca sexta* [69].

#### Sigma GSTs

Sigma GSTs play a protective role upon exposure to oxidative stress [70,71]. Likewise, in *A*. *mellifera* (4 Sigma class GST members), this class is the most abundant among the triatomine GST superfamily with 7 genes in *R*. *prolixus*, 9 in *T*. *infestans*, 13 in *T*. *dimidiata* and 5 in *T*. *pallidipennis* (Table 1) (see Supplementary S1 File for the analysis of transmembrane domain and subcellular localization of Sigma GSTs). In contrast, this class is reduced in *D*. *melanogaster* and *An*. *gambiae* with only one member, while *A*. *pisum* has 6 genes (Table 1). Within the *R*. *prolixus* GST superfamily, the only case of gene duplication would be represented by the RPRC011244 and RPRC011128 Sigma GST genes, which are located in supercontig KQ034372. The higher representation of Sigma GSTs in triatomines could reflect a role in coping with the oxidative stress generated by the hydrolysis of hemoglobin and the release of cytotoxic heme molecules after blood feeding [72].

Concerning the transcriptomic studies, all Sigma GSTs were highly expressed in *R*. *prolixus* antennae, except for RPRC010609 and RPRC011244 (Fig 6). The latter was highly expressed in fat body, Malpighian tubules, rectum and ovary (Supplementary S5 File). Sigma class GSTs did not show differences between stages or sexes in the antennal transcriptomes (Fig 6). In the CNS, the GST genes with the highest expression were RPRC010609, RPRC015269 and RRPC007353 (Fig 6), the latter being also highly expressed in digestive structures and gonads (Supplementary S5 File).

#### Omega, Theta and Zeta classes

Omega GSTs, important in the removal of S-thiol from proteins [73], have not been detected in *T*. *infestans* whereas they are represented by only 1 gene in *T*. *pallidipennis* and *R*. *prolixus*. The expression of RPRC007741 was detected in antennae (Fig 6) and in digestive structures and gonads at relatively high levels (Supplementary S5 File). In *T*. *dimidiata*, 2 Omega GST genes were found. This is similar to what has been observed in *A*. *mellifera*, *A*. *pisum* and *An*. *gambiae* (Table 1). The Omega class is comparatively expanded in *D*. *melanogaster*, which presents 4 transcripts (Table 1).

A function in the metabolism of endogenous substrates has not yet been assigned for Theta class GSTs, although their contribution to the detoxification of xenobiotics has been proposed [40]. Theta class GSTs are expanded in *D*. *melanogaster* (4 transcripts) compared with *A*. *mellifera* (1 transcript), *An*. *gambiae* (2 transcripts), and *A*. *pisum* (2 transcripts). Theta GST class presented differences across the triatomine species analyzed here. Whereas *R*. *prolixus* possesses 3 predicted transcripts of this class, we detected 2 transcripts in *T*. *dimidiata* and *T*. *infestans*, and only 1 in *T*. *pallidipennis*. The genes RPRC009617 and RPRC009358 were expressed in antennae and the CNS (Fig 6), whereas the expression of RPRC009138 was high in digestive structures and gonads (Supplementary S5 File).

Finally, the Zeta GST class, involved in the degradation of tyrosine and phenylalanine, was represented by one gene in each triatomine species except for *T*. *infestans* where it was not detected (Table 1). Similarly, *A*. *mellifera* and *An*. *gambiae* also have one Zeta class gene, while *D*. *melanogaster* has 2 of these genes and they are absent in *A*. *pisum* (Table 1 and Fig 5). The Zeta class gene was highly expressed in the CNS, antennae (Fig 6), digestive structures and gonads (Supplementary S5 File).

### Gene expression comparisons between sensitive and resistant populations of *T*. *infestans*

**Fig 7:**
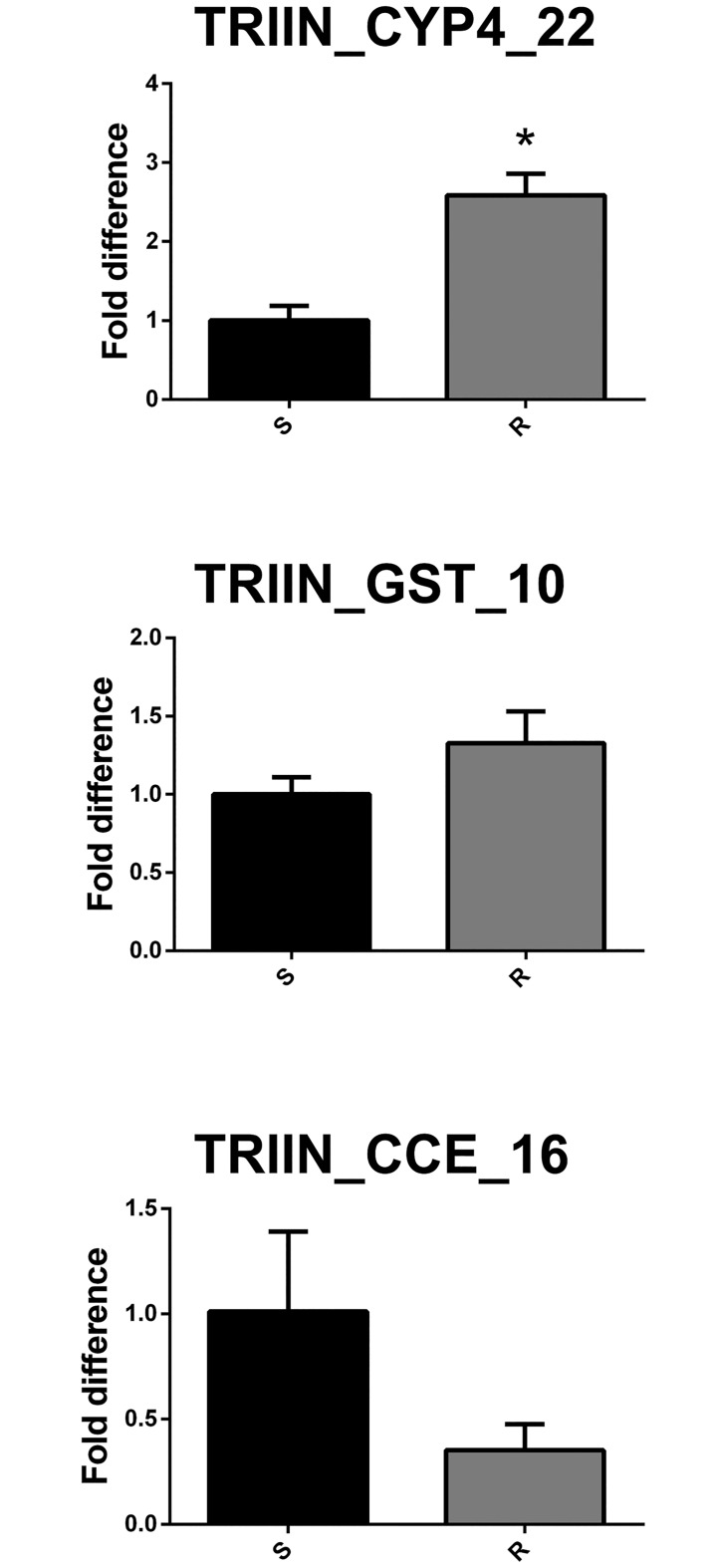
Gene expression analysis of detoxification-related genes in *T*. *infestans* from a sensitive laboratory (S) and a resistant (R) population. Results are expressed as the mean ± S.E (n = 4/group) of the fold difference respect to average for the sensitive population. * = p<0.05.

The expression levels of three insecticide-resistance related genes were compared between pyrethroid sensitive and resistant populations of *T*. *infestans*. For these comparisons we chose three genes belonging to each one of the superfamilies under study. Within each superfamily, we chose to test a gene belonging to a clade related to insecticide resistance (CYP4, Clade E CCE and Delta GST). Interestingly, we found that the CYP4 gene analyzed was significantly overexpressed in insects belonging to the resistant population (p< 0.05; n = 4) (Fig 7). Overexpression of a CYP4 gene in a resistant *T*. *infestans* population was recently reported [13], suggesting that this could be an expanded mechanism of pyrethroid resistance in this species. The expression of the other enzymes studied did not significantly differ between sensitive and resistant populations (Fig 7).

## Concluding remarks

The analysis of three *Triatoma* spp. transcriptomes and the *R*. *prolixus* genome and transcriptomes allowed us to present a functional overview of the three main insecticide detoxification-related enzyme superfamilies found in vectors of Chagas’ disease. Overall, when triatomines were compared with other insect species, the results point to a reduction in some enzyme clades associated to xenobiotic detoxification: absence of Epsilon class and reduced Delta class in GST superfamily; absence of mitochondrial clade CYP12; absence of CYP9 family in CYP3 clade; and the absence of dietary class CCEs. Conversely, other detoxification-related families are expanded in triatomines: CYP3 clade, clade E in the CCE superfamily and Sigma Class GST. From our point of view, those clades present the main detoxification potential of all triatomine species analyzed here. The overexpression of a CYP4 gene found in a *T*. *infestans* resistant population suggests that this family would also represent a detoxification mechanism in this insect species. Besides, most of the recently described CYP families within the CYP3 and CYP4 clades in *R*. *prolixus* were confirmed in *Triatoma* spp. The high abundance of some members in these triatomine specific families makes them candidates to perform physiological and genetic studies.

Overall, our findings contribute to understanding insecticide resistance in vectors of Chagas' disease, and open up possibilities for functional studies on triatomine detoxification mechanisms.

## Supporting information

S1 File Structural analysis of CYP4 (tab1), CYP3 (tab2), GST (tab3) and clade E of CCE (tab4) sequences of *R*. *prolixus* and *Triatoma* spp.; and relation between the contig codes and the assigned names of *Triatoma* spp. transcripts (tab5) used in Figs 1, 3 and 5 (main text). The structural analysis includes the prediction of number and position for the transmembrane domains, the prediction of the subcellular localization and the MOTIF search (http://www.genome.jp/tools/motif/MOTIF.html) using the Conserved Domain Database (CDD) and the Pfam database, and includes the representative domain for each superfamily, its localization in the protein sequence and E-value.
(XLSX)

S2 File *Triatoma* spp. protein sequences used to construct the phylogenetic trees. (TXT)

S3 File Reference and target transcripts used in qRT-PCR experiments, their primer sequences and amplicon length. (DOCX)

S4 File Phylogenies of the mitochondrial CYP clade (Fig 1a), CYP2 clade (Fig 1b), CYP3 clade (Fig 1c), CYP4 clade (Fig 1d), CCE superfamily (Fig 2) and Glutathione Transferase superfamily (Fig 3) from *R*. *prolixus*, *Triatoma* spp., *D*. *melanogaster*, *An*. *gambiae*, *A*. *mellifera*, *A*. *pisum*, *T*. *castaneum* and *B*. *mori*. (DOCX)

S5 File Expression of GST, CYP and CCE superfamilies in *R*. *prolixus* tissue-specific transcriptomes. The expression of each transcript in the different transcriptomes (anterior and posterior midgut, hindgut, Malpighian tubules, fat body, gonads and whole body) was represented as number of mapped reads. These *R*. *prolixus* tissue-specific transcriptomes were constructed by Oliveira *et al*. and their sequencing output is publicly available at http://rhodnius.iq.ufrj.br.
(XLSX)
