# Supplementary material for: Comparative and functional triatomine genomics reveals reductions and expansions in insecticide resistance-related gene families
Source: PLoS Negl Trop Dis. 2017 Feb 15;11(2):e0005313. doi: 10.1371/journal.pntd.0005313 (PMC5310753; doi:10.1371/journal.pntd.0005313)
Supplement: S3 File — (DOCX) [file pntd.0005313.s003.docx]

| **Transcript** | **Primer forward (5´-3´)** | **Primer reverse (5´-3´)** | **Amplicon length (bp)** |
| --- | --- | --- | --- |
| *β-Actin* | CTCTGGCAGAACAACTGGTATC | GCCAAGTCCAAACGAAGAATTG | 111 |
| *α-Tubulin* | CAGACTGTGCTTTCATGGTAGA | CTGCCCTATCAGTCTGTTCAAG | 107 |
| *G6PDH* | GGGACGTGGCGGTTATTT | TTTCCATGGCAACAAGACTTA | 92 |
| *TRIINF_GST_10* | GCCGTGCTATTGCAACTTAC | CTGATCCACGATGGCTTTCT | 92 |
| *TRIIN_CYP4_22* | CGTACGCCTATATACCATTCAGC | CTCAGTATTGTAGAGAGGACAACTTT | 97 |
| *TRIINF_CCE_16* | CCAGAATACGTTACGGTAAACCTC | GTTGCATCTAATACTTTCTTCCAAGG | 91 |
