# Supplementary material for: Comparative and functional triatomine genomics reveals reductions and expansions in insecticide resistance-related gene families
Source: PLoS Negl Trop Dis. 2017 Feb 15;11(2):e0005313. doi: 10.1371/journal.pntd.0005313 (PMC5310753; doi:10.1371/journal.pntd.0005313)
Supplement: S4 File — (DOCX) [file pntd.0005313.s004.docx]

**Figure 1a**. Phylogeny of the mitochondrial CYP clade from *R. prolixus* (VectorBase ID shown), *T. infestans* (TINF), *T. dimidiata* (TDIM), *T. pallidipennis* (TPAL), *Drosophila melanogaster* (DROME; <http://flybase.org/>), *Anopheles gambiae* (ANOGA; sequences obtained from <https://www.vectorbase.org/>), *Apis mellifera* (APIME; sequences obtained from <http://hymenopteragenome.org/beebase/>, described by Claudianos *et al.,* 2006) and *Acyrtosiphon pisum* (ACYPI; only sequences available at NCBI described by Ramsey *et al.,* 2010 were included). The sequence of Neurotactin from *D.* melanogaster (CG9704) was used as outgroup. The triatomine sequences are painted in grey. The relation between the contig code and the assigned name (used in Figure 1 of the main text) is showed in Supplementary file 1 (Tab 5).

**Figure 1b**. Phylogeny of the CYP2 clade from *R. prolixus* (VectorBase ID shown), *T. infestans* (TINF), *T. dimidiata* (TDIM), *T. pallidipennis* (TPAL), *Drosophila melanogaster* (DROME; sequences obtained from <http://flybase.org/>), *Anopheles gambiae* (ANOGA; sequences obtained from <https://www.vectorbase.org/>), *Apis mellifera* (APIME; sequences obtained from <http://hymenopteragenome.org/beebase/> and described by Claudianos *et al.,* 2006) and *Acyrtosiphon pisum* (ACYPI; only available sequences at NCBI described by Ramsey *et al.,* 2010 were included). The sequence of Neurotactin from *D. melanogaster* (CG9704) was used as outgroup. The triatomine sequences are painted in grey. The relation between the contig code and the assigned name (used in Figure 1 of the main text) is showed in Supplementary file 1 (Tab 5). The sequence TRIPA_H9TUR5Q02F8MDG_222_341_r, which was not classified in Figure 1 (main text), was identified as CYP305 in this analysis. This ambiguous classification could be explained by its short length.

**Figure 1c**. Phylogeny of the CYP3 clade from *R. prolixus* (VectorBase ID shown), *T. infestans* (TINF), *T. dimidiata* (TDIM), *T. pallidipennis* (TPAL), *Drosophila melanogaster* (DROME; sequences obtained from <http://flybase.org/>), *Anopheles gambiae* (ANOGA; sequences obtained from <https://www.vectorbase.org/>), *Apis mellifera* (APIME; sequences obtained from <http://hymenopteragenome.org/beebase/> and described by Claudianos *et al.,* 2006) and *Acyrtosiphon pisum* (ACYPI; only sequences available at NCBI described by Ramsey *et al,.* 2010 were included). The sequence of Neurotactin from *D. melanogaster* (CG9704) was used as outgroup. The triatomine sequences are painted in grey. The relation between the contig code and the assigned name (used in Figure 1 of the main text) is showed in Supplementary file 1 (Tab 5). The sequence TRIDI_H9TUR5Q02J0EHK_513_608, which was classified as CYP3084 in Figure 1 (main text), was identified as CYP3089 in this analysis. This ambiguous classification could be explained by its short length.

**Figure 1d**. Phylogeny of the CYP4 clade from *R. prolixus* (VectorBase ID shown), *T. infestans* (TINF), *T. dimidiata* (TDIM), *T. pallidipennis* (TPAL), *Drosophila melanogaster* (DROME; sequences obtained from <http://flybase.org/>), *Anopheles gambiae* (ANOGA; sequences obtained from <https://www.vectorbase.org/>), *Apis mellifera* (APIME; sequences obtained from <http://hymenopteragenome.org/beebase/> and described by Claudianos *et al.,* 2006) and *Acyrtosiphon pisum* (ACYPI; only sequences available at NCBI described by Ramsey *et al.,* 2010 were included). The sequence of Neurotactin from *D. melanogaster* (CG9704) was used as outgroup. The triatomine sequences are painted in grey. The relation between the contig code and the assigned name (used in Figure 1 of the main text) is showed in Supplementary file 1 (Tab 5).

**Figure 2**. Phylogeny of the CCE superfamily from *R. prolixus* (VectorBase ID shown), *T. infestans* (TINF), *T. dimidiata* (TDIM), *T. pallidipennis* (TPAL), *Drosophila melanogaster* (DROME; sequences obtained from <http://flybase.org/> and described by Oakeshott *et al.,* 2005 and 2010), *Anopheles gambiae* (ANOGA; sequences obtained from <https://www.vectorbase.org/> and NCBI described by Oakeshott *et al.,* 2005 and 2010), *Apis mellifera* (APIME; sequences obtained from <http://hymenopteragenome.org/beebase/> and described by Claudianos *et al.,* 2006), *Tribolium castaneum* (TRICA; sequences obtained from <http://beetlebase.org/> and described in Oakeshott *et al.,* 2010) and *Acyrtosiphon pisum* (ACYPI; only sequences available at NCBI described by Ramsey *et al.,* 2010 were included). The sequence of *Cyp4c3* from *D. melanogaster* (CG14031) was used as outgroup. The letters depicted next to the dots in the branches of the tree indicate the delimitation of each clade. The relation between the contig code and the assigned name (used in Figure 1 of the main text) is showed in Supplementary file 1 (Tab 5). The sequences TDIM_IAZY42G01ETAQS_3_395, TDIM_IAZY42G01DFMVR_26_397, TDIM_H9TUR5Q01EPXYH_1_465 and TPAL_isotig05701_255_611, which were classified as Clade E CCEs in Figure 1 (main text), appear as unclassified in this analysis. This ambiguous classification could be explained by their short length.

**Figure 3**. Phylogeny of the Glutathione Transferase superfamily from *R. prolixus* (VectorBase ID shown), *T. infestans* (TINF), *T. dimidiata* (TDIM), *T. pallidipennis* (TPAL), *Drosophila melanogaster* (DROME), *Anopheles gambiae* (ANOGA), *Apis mellifera* (APIME), *Acyrtosiphon pisum* (ACYPI), *Tribolium castaneum* (TRICA) and *Bombyx mori* (BOMMO). The information about these sequences is available in Shi *et al.,* (2012). The sequence of *Cyp4c3* from *D. melanogaster* (CG14031) was used as outgroup. The relation between the contig code and the assigned name (used in Figure 1 of the main text) is showed in Supplementary file 1 (Tab 5). The sequence TDIM_IAZY42G02GI5D9_78_278, which was classified as Sigma in Figure 5, was identified as a microsomal GST in this analysis. This ambiguous classification could be explained by its short length.
